# Intrahepatic transcriptomics reveals gene signatures in chronic hepatitis B patients responded to interferon therapy

**DOI:** 10.1080/22221751.2022.2100831

**Published:** 2022-07-27

**Authors:** Ning Li, Kangkang Yu, Minhui Dong, Jinyu Wang, Feifei Yang, Haoxiang Zhu, Jie Yu, Jingshu Yang, Wentao Xie, Bidisha Mitra, Richeng Mao, Feizhen Wu, Haitao Guo, Jiming Zhang

**Affiliations:** a Department of Infectious Diseases, Shanghai Key Laboratory of Infectious Diseases and Biosafety Emergency Response, Shanghai Institute of Infectious Diseases and Biosecurity, National Medical Center for Infectious Diseases, Huashan Hospital, Fudan University, Shanghai, People’s Republic of China; b Key Laboratory of Medical Molecular Virology (MOE/MOH), Shanghai Medical College, Fudan University, Shanghai, People’s Republic of China; c Cancer Virology Program, UPMC Hillman Cancer Center, Department of Microbiology and Molecular Genetics, University of Pittsburgh, Pittsburgh, PA, USA; d Laboratory of Epigenetics, Institutes of Biomedical Sciences, Fudan University, Shanghai, People's Republic of China; e Key Laboratory of Birth Defects, Children's Hospital of Fudan University, Shanghai, People's Republic of China

**Keywords:** Chronic hepatitis B, interferon therapy, liver biopsy, transcriptome, HBeAg

## Abstract

Chronic hepatitis B virus (HBV) infection remains a substantial public health burden worldwide. Alpha-interferon (IFNα) is one of the two currently approved therapies for chronic hepatitis B (CHB), to explore the mechanisms underlying IFNα treatment response, we investigated baseline and 24-week on-treatment intrahepatic gene expression profiles in 21 CHB patients by mRNA-seq. The data analyses demonstrated that PegIFNα treatment significantly induced antiviral responses. Responders who achieved HBV DNA loss and HBeAg or HBsAg seroconversion displayed higher fold change and larger number of up-regulated interferon-stimulated genes (ISGs). Interestingly, lower expression levels of certain ISGs were observed in responders in their baseline biopsy samples. In HBeAg+ patients, non-responders had relative higher baseline HBeAg levels than responders. More importantly, HBeAg− patients showed higher HBsAg loss rate than HBeAg+ patients. Although a greater fold change of ISGs was observed in HBeAg− patients than HBeAg+ patients, upregulation of ISGs in HBeAg+ responders exceeded HBeAg− responders. Notably, PegIFNα treatment increased monocyte and mast cell infiltration, but decreased CD8 T cell and M1 macrophage infiltration in both responders and non-responders, while B cell infiltration was increased only in responders. Moreover, co-expression analysis identified ribosomal proteins as critical players in antiviral response. The data also indicate that IFNα may influence the production of viral antigens associated with endoplasmic reticulum. Collectively, the intrahepatic transcriptome analyses in this study enriched our understanding of IFN-mediated antiviral effects in CHB patients and provided novel insights into the development of potential strategies to improve IFNα therapy.

## Introduction

Hepatitis B virus (HBV) infection remains a severe public health threat worldwide, with more than 250 million chronically infected individuals at increased risk of developing advanced liver disease and hepatocellular carcinoma, which caused an annual death of more than 860,000 people [1]. Alpha-interferon (IFNα) and nucleos(t)ide analogues are the two currently approved therapies for chronic hepatitis B (CHB) [2]. With both antiviral and immunomodulatory effects, IFNα therapy can induce a higher durable HBsAg loss rate, but viral response to IFNα is limited to a minor portion of patients and the underlying mechanism remains elusive [3].

It is well acknowledged that CHB is a consequence of immune response against HBV, instead of cytopathic effects [4]. In CHB, IFNα generally exhibits its anti-HBV effects by either inducing interferon-stimulated genes (ISGs) or coordinating the innate and adaptive immune responses [5]. High pretreatment expression of ISG in liver tissues of chronic hepatitis C patients impedes the response to IFNα therapy [6], similar findings were observed in CHB patients as well [7]. It has even been previously reported that IFNα can induce sustained antiviral response without immune cell involvement [8]. However, a study on the woodchuck model of CHB suggested that the antiviral response by IFNα is associated with NK/T cell and B cell signatures, beyond the induction of antiviral ISGs [9]. Other clinical studies reported that IFNα treatment led to significant expansion of natural killer (NK) cells and subsequent IFN-γ production [10], and that IFN-γ and B cell signatures were correlated with IFNα treatment response [11]. In addition, high pretreatment expression of genes involved in adaptive immunity was found to be associated with favourable IFNα treatment response [12]. Although these studies have enriched our understanding of the anti-HBV effects of IFNα, but the discrepant findings prompted us to conduct further investigation.

Fine needle aspirates are capable of providing comprehensive landscape of intrahepatic immunity [13]. Given that 24 weeks after PegIFNα treatment is a critical time point to evaluate on-treatment response [14], in the current study, we aim to investigate the intrahepatic gene expression profiles of CHB patients by fine needle aspiration before and after 24 weeks PegIFNα therapy, thereby evaluating the effects of IFNα on ISG expression, innate and adaptive immune responses. More importantly, by comparing transcriptional signatures between responders and non-responders, we intend to determine whether pre-treatment expression levels or on-treatment alterations of certain gene expression are predictive of a favourable response to IFNα treatment. The impact of HBeAg status on PegIFNα treatment response will be evaluated as well.

## Materials and methods

### Study population

Treatment-naïve CHB patients who received baseline liver biopsy within 3 weeks before starting PegIFNα therapy were enrolled. A second liver biopsy was performed post 24-week PegIFNα treatment. In the present study, a responder was defined as a patient who achieved undetectable HBV DNA as well as HBsAg and/or HBeAg seroconversion during a 48-week follow-up post 48 weeks’ PegIFNα therapy. Clinical data was retrieved from the medical record system. All patients provided written informed consent and the protocol was approved by the Institutional Review Board of Huashan Hospital, Fudan University.

### Statistical analysis

Comparison between groups of patients was performed using Student’s *t*-test or the non-parametric Mann–Whitney *U* test. *p* value <0.05 is considered to be statistically significant.

(Additional materials and methods are available in the Supplemental Material.)

## Results

### Study design and clinical characteristics of patients

**Figure 1.**
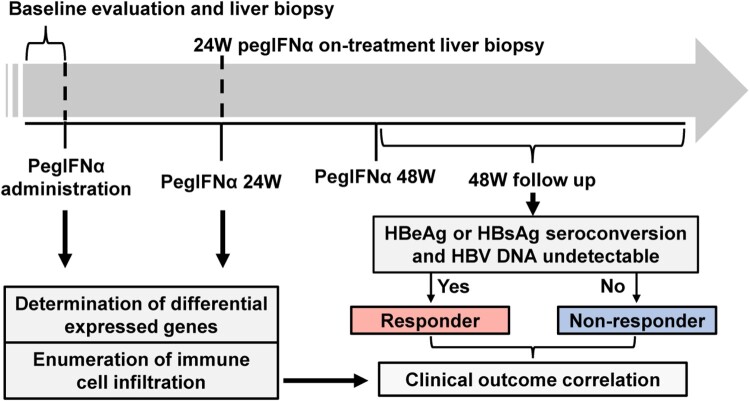
Flow diagram of the study design. Patients received PegIFNα treatment for 48 weeks and followed up for another 48 weeks post-treatment. Liver biopsies were performed before treatment and 24-week on-treatment. Biopsy samples were subjected to mRNA-seq and analysed to evaluate gene expression and immune cell infiltration levels according to patients’ treatment response.

Twenty-one treatment-naïve CHB patients (16 males and 5 females) who were treated weekly with PegIFNα were enrolled in this study. Each patient received two liver biopsies, i.e. the pretreatment baseline biopsy and the on-treatment biopsy obtained at week 24 of IFN treatment (Figure 1). The demographic and clinical characteristics of the included patients are shown in Supporting Table S1. Eight patients who achieved HBV DNA undetectable (<50 IU/mL) and underwent either HBeAg seroconversion (HBeAg positive at baseline) or HBsAg seroconversion (HBeAg negative at baseline) at the end of the treatment or during 48-week follow-up were defined as responders, whereas the remaining 13 patients were classified as non-responders.

### Global hepatic gene expression alteration in response to PegIFNα

**Figure 2.**
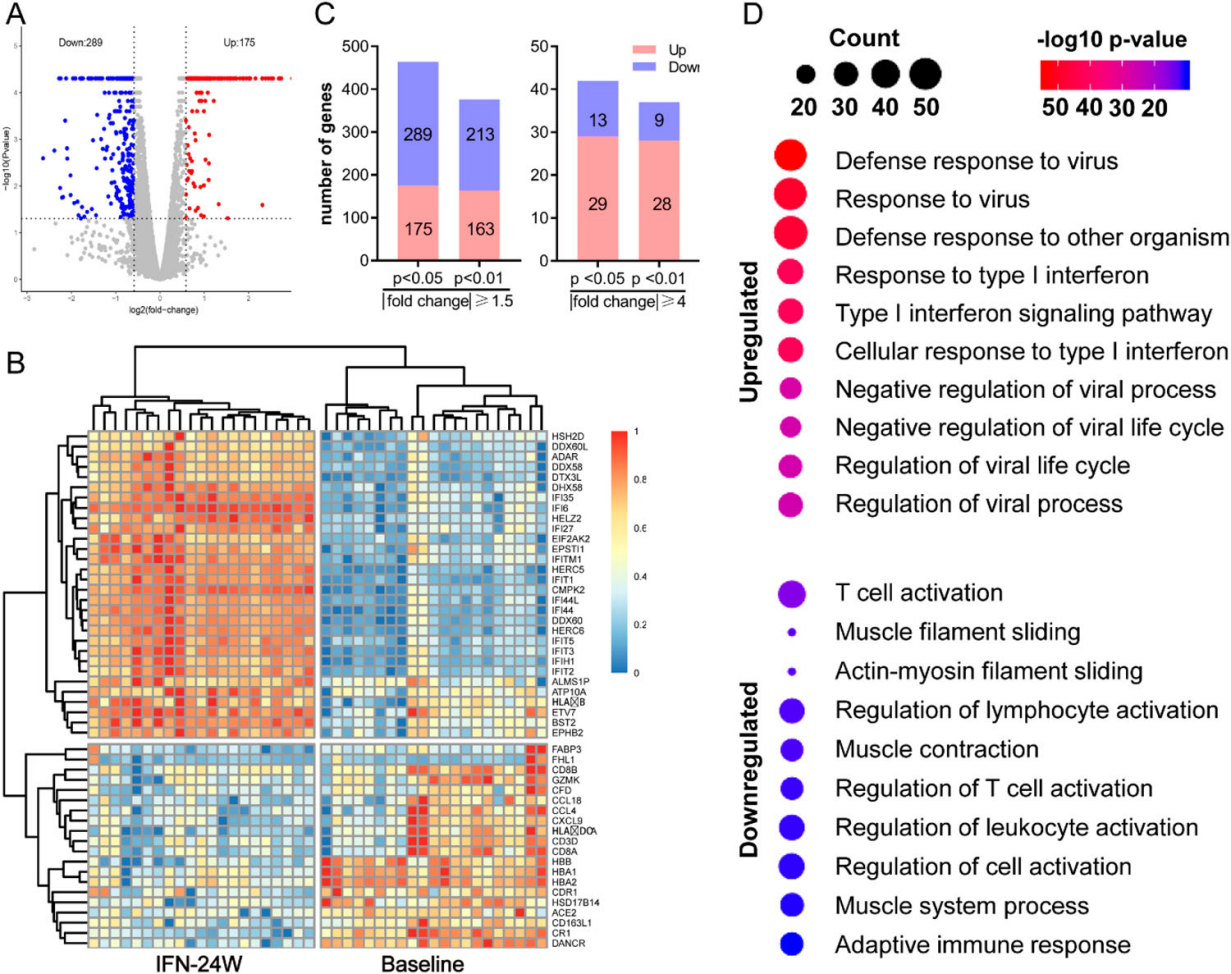
PegIFNα treatment activates antiviral response. (A) Gene expression levels at baseline and 24-week on-treatment were compared, which identified 175 up-regulated and 289 down-regulated genes (*p* < 0.05 and |fold change| ≥1.5). (B) The top 50 (30 up-regulated and 20 down-regulated) differentially expressed genes were presented. (C) More genes down-regulated than up-regulated at the threshold of fold change| ≥1.5 and *p* <0.05 or 0.01, when |fold change| ≥4, up-regulated genes outnumbered down-regulated genes. (D) Functional enrichment analysis revealed that the 175 up-regulated and 289 down-regulated genes were enriched in antiviral defense responses and T cell activation, respectively.

To reveal the impact of PegIFNα on intrahepatic gene expression, we performed mRNA-seq of collected liver biopsy samples. Overall, 464 genes (175 up-regulated and 289 down-regulated) with absolute fold change ≥1.5 and *p* value <0.05 in response to 24 weeks PegIFNα therapy were identified (Figure 2(A)). Heatmap shows the top 50 (30 up-regulated and 20 down-regulated) most significantly differentially expressed genes (DEGs), which includes members of the retinoic acid-inducible gene (RIG)-I-like receptor (RLR) family-DDX58/RIG-I, DDX60 and IFIH1 (Figure 2(B)). These genes are important pattern recognition receptors involving in the recognition of viruses by the innate immune system [15,16]. The upregulation of antiviral defense related genes by multiple folds was an interesting observation made, indicating that the most significant DEGs are predominantly the up-regulated ones (Figure 2(C)). The total DEGs were then subjected to functional enrichment analysis, which revealed that the up-regulated genes were mainly involved in response to IFNα and defense response to virus, while the down-regulated genes were predominantly implicated in leukocyte activation, including T cell activation (Figure 2(D)).

### Gene set enrichment analysis

**Figure 3.**
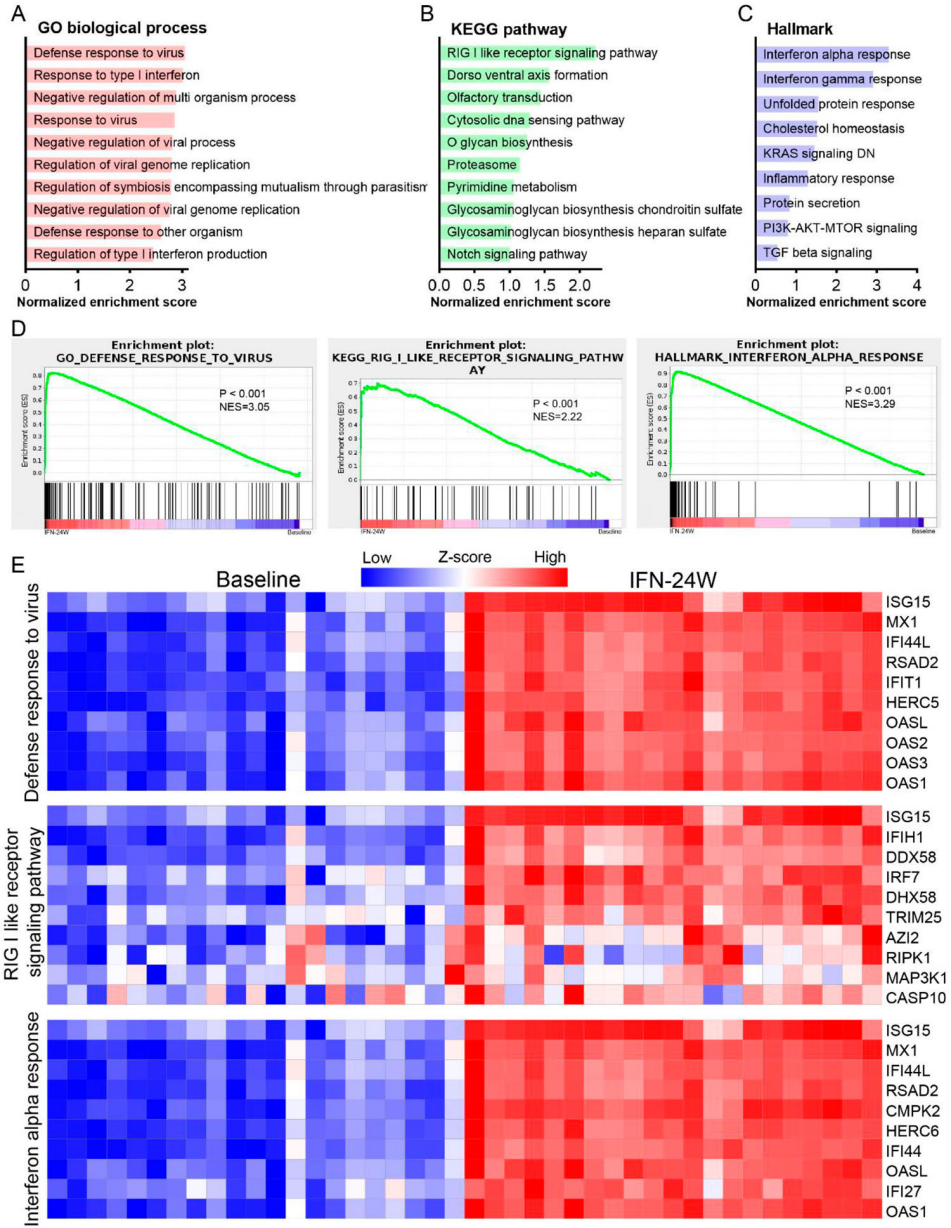
Gene set enrichment analysis. (A) Significantly enriched GO biological processes were mainly involved in antiviral responses. (B) The RIG-I-like receptor pathway was the most significantly enriched KEGG pathway. (C) Interferon alpha response and interferon gamma response were the most enriched Hallmarks. (D) The GSEA plot of the most enriched GO biological process (defense response to virus), KEGG pathway (RIG-I-like receptor pathway) and Hallmark (Interferon alpha response). (E) Heatmap demonstrated expressions of the top 10 genes in each of the most enriched gene sets.

We next performed gene set enrichment analysis (GSEA) to examine which pathways are correlated with PegIFNα administration. There were 718, 9, and 28 gene sets that were enriched for Gene Ontology (GO) biological processes, Hallmark annotated by GSEA and KEGG pathways, respectively. The results demonstrated that these top gene sets were significantly related to defense response to virus. In detail, the most enriched top 10 GO biological processes were all involved in response to IFNα or negative regulation of viral process (Figure 3(A)). The most significantly enriched KEGG pathway was RIG-I like receptor signalling pathway (Figure 3(B)) and the most significantly enriched hallmark pathway was IFNα response (Figure 3(C)). Representative enrichment plots of the most enriched gene set in each category were displayed (Figure 3(D)), and heatmap shows expression alteration of the top 10 genes of each gene set (Figure 3(E)).

### ISG expression analysis

**Figure 4.**
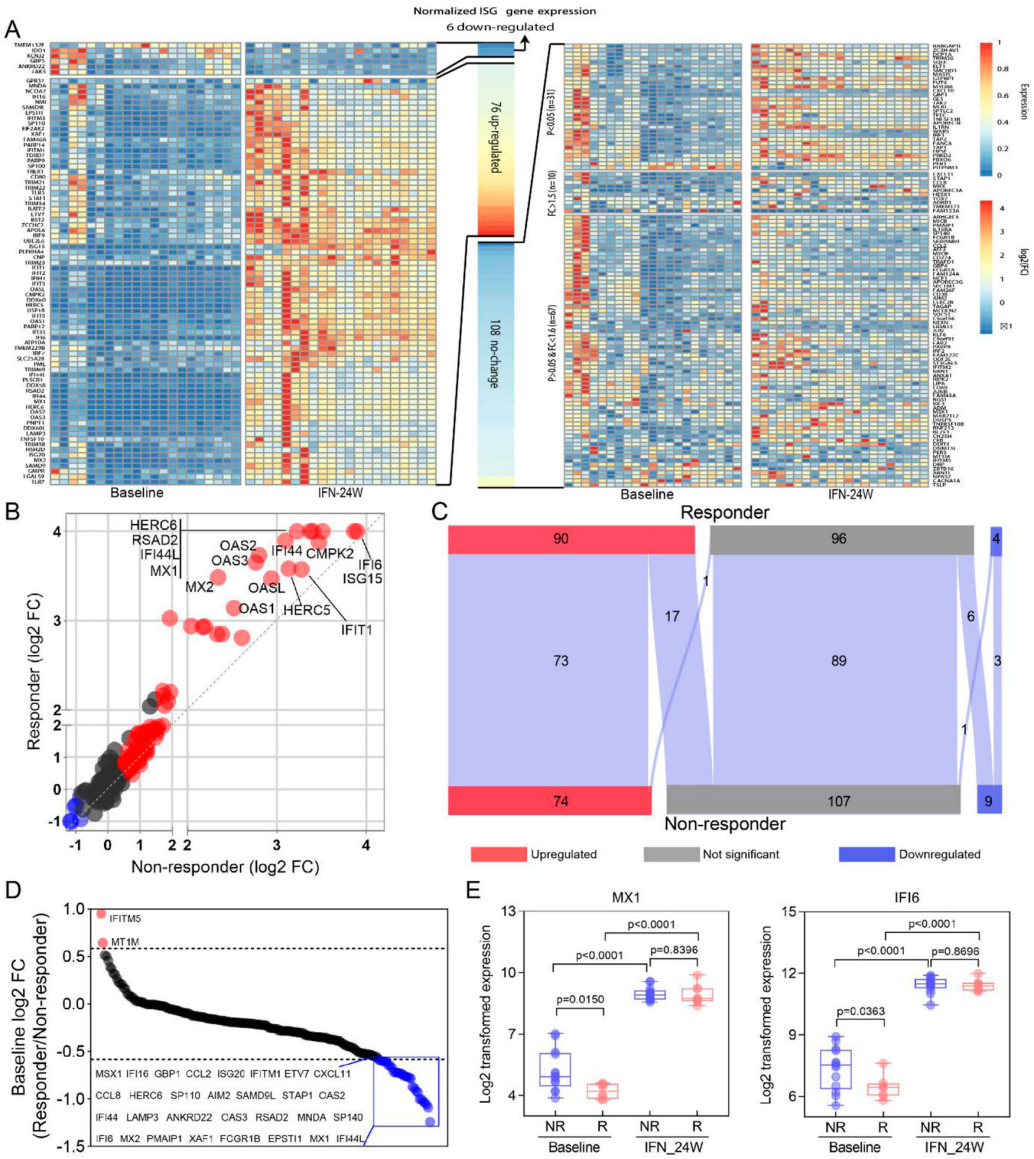
ISG expression analysis. (A) Heatmap displayed expression alteration of the 190 ISGs in general population between IFN-24W and baseline. The results showed that 76 ISGs were up-regulated and 6 ISGs were down-regulated after 24-week PegIFNα treatment, while alteration of the remaining 108 ISGs was not statistically significant. (B) ISGs expression alteration analysed separately in responders and non-responders revealed that most ISGs up-regulated higher in the responder. (C) Summary of ISGs expression alteration in responders and non-responders and their relationships. (D) Expression comparison revealed that 30 ISGs expressed lower in responders at baseline. (E) Boxplot of ISG (MX1 and IFI6) expression at baseline and IFN-24W showed higher fold change in responders rather than non-responders, since comparable expression levels at IFN-24W but lower expression levels in responders at baseline were observed. R, responder; NR, non-responder; FC, fold change.

It is well known that IFN exerts its antiviral effects intracellularly by regulating the expression of ISGs [17]. To evaluate the impact of IFNα treatment on ISG expression, a list of 190 IFN-induced genes collected from published literatures was analysed [6,18], and the results showed that 76 genes were up-regulated, 6 genes were down-regulated, and 108 were unchanged with the threshold of absolute fold change ≥1.5 and *p* <0.05 (Figure 4(A)). As expected, many critical antiviral ISGs, such as DDX58, DDX60, IFI44, IFI6, ISG15, ISG20, MX2 and OAS1/2/3 appeared in the up-regulated genes. To explore the relationship between intrahepatic ISG expression and treatment response, we analysed expression alteration of the 190 genes in responders and non-responders separately. The results showed that most ISGs were up-regulated more significantly in responders (Figure 4(B)). Generally, 90 genes were up-regulated, 4 genes were down-regulated, and 96 genes remain unchanged in the responder group; while in the non-responder group, the number was 74, 9 and 107, respectively (Figure 4(C)). We then compared baseline and IFN-24W levels of these ISGs and found that 30 ISGs exhibited lower baseline expression levels in responders, while their expression levels at IFN-24W were comparable (Figure 4(D); Supporting Fig. S1A). Expressions of MX1 and IFI6 were displayed as representatives (Figure 4(E,F)). To date, a number of ISGs have been identified as suppressor of HBV replication (Supporting Table S2). The differential expressions of selected HBV suppressor ISGs in responders and non-responders at baseline and IFN-24W are displayed in Supporting Fig. S1B.

### Identification of genes related to PegIFNα treatment response

**Figure 5.**
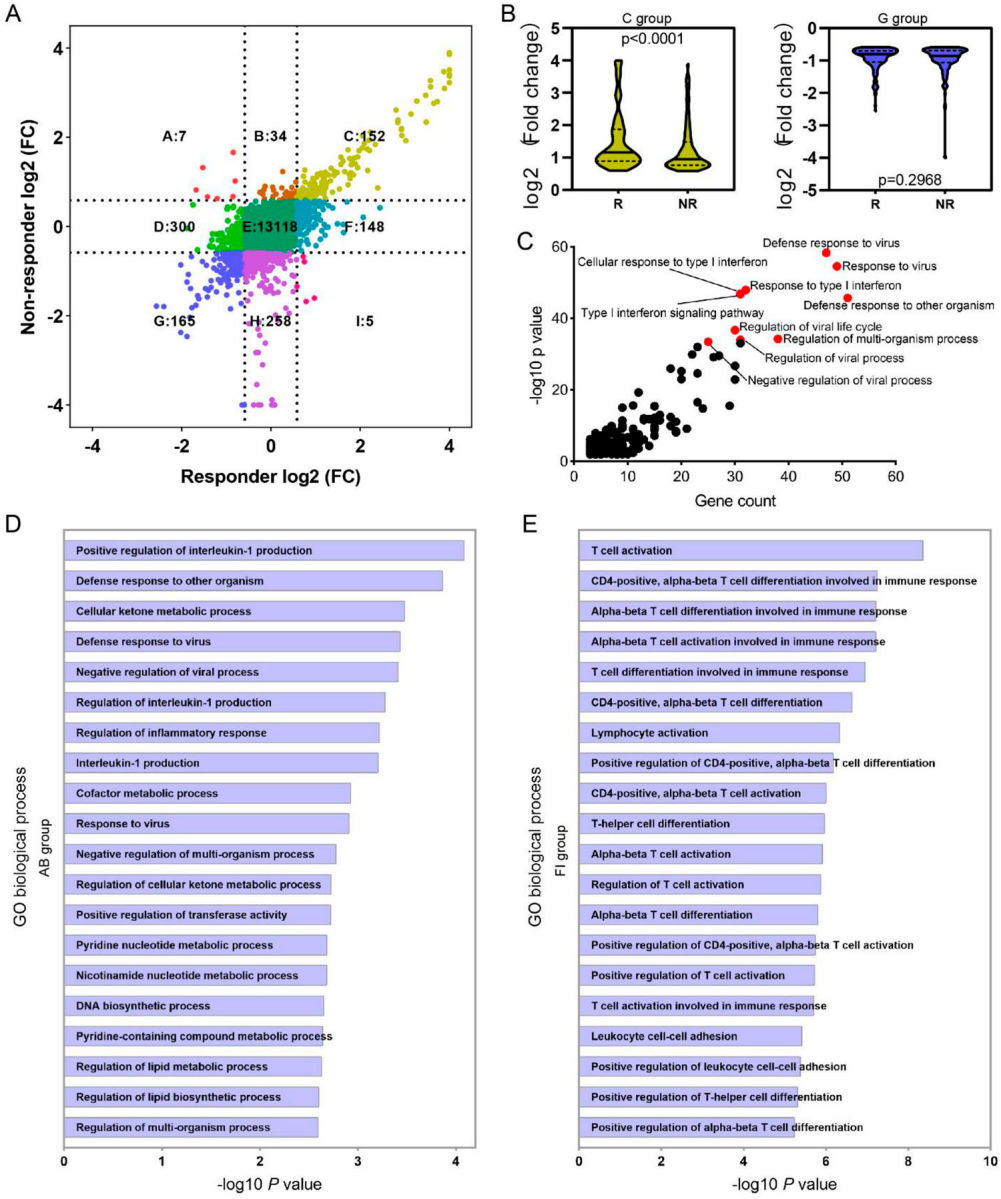
Identification of genes related to PegIFNα treatment response. (A) Alteration of gene expression was analysed separately in responders and non-responders, 41 genes (group AB) were up-regulated in the non-responder group but down-regulated (*n* = 7) or not changed (*n* = 34) in the responder group, 152 genes (group C) up-regulated both in responders and non-responders, 153 genes (group FI) were up-regulated in the responder group but down-regulated (*n* = 5) or not changed (*n* = 148) in the non-responder group, 165 genes (group G) down-regulated both in responders and non-responders. (B) Fold change comparison between responders and non-responders revealed that genes in group C up-regulated more significantly in responders, while down-regulated genes in group G were comparable. (C) GO term enrichment analysis showed that genes in group C had significantly enriched antiviral signalling, including defense response to virus, response to type I IFN. (D) Enrichment analysis revealed that genes in group AB were mainly implicated in regulation of interleukin-1 production and defense response. (E) Enrichment analysis of genes in group FI demonstrated that the most enriched biological processes were T cell activation and differentiation. R, responder; NR, non-responder; FC, fold change.

The difference of ISG expression between responders and non-responders prompted us to explore gene expression pattern at a global level, and thus total genes were classified into nine groups according to their expression alteration (Figure 5(A)). For genes up-regulated in both responders and non-responders (C group), a relative higher fold change was observed in responders (Figure 5(B)); while for genes down-regulated in both responders and non-responders (G group), no significant fold change difference was found (Figure 5(B)). Functional enrichment analysis of genes in the C group revealed that these genes were mainly implicated in defense response to virus and Type I IFN signalling pathway (Figure 5(C)). In addition, 41 genes up-regulated in non-responders (A and B groups) were either showed no change (n = 34) or even down-regulated (n = 7) in responders (Figure 5(A)). GO enrichment analysis showed that these 41 genes were enriched in Interleukin-1 production regulation and defense response to virus (Figure 5(D)). While 153 genes up-regulated in responders (F and I groups) either showed no change (n = 148) or even down-regulated (n = 5) in non-responders (Figure 5(A)). Functional enrichment analysis revealed that these genes mainly participate in T cell activation and differentiation during adaptive immune response (Figure 5(E)).

### Immune cell infiltration analysis

**Figure 6.**
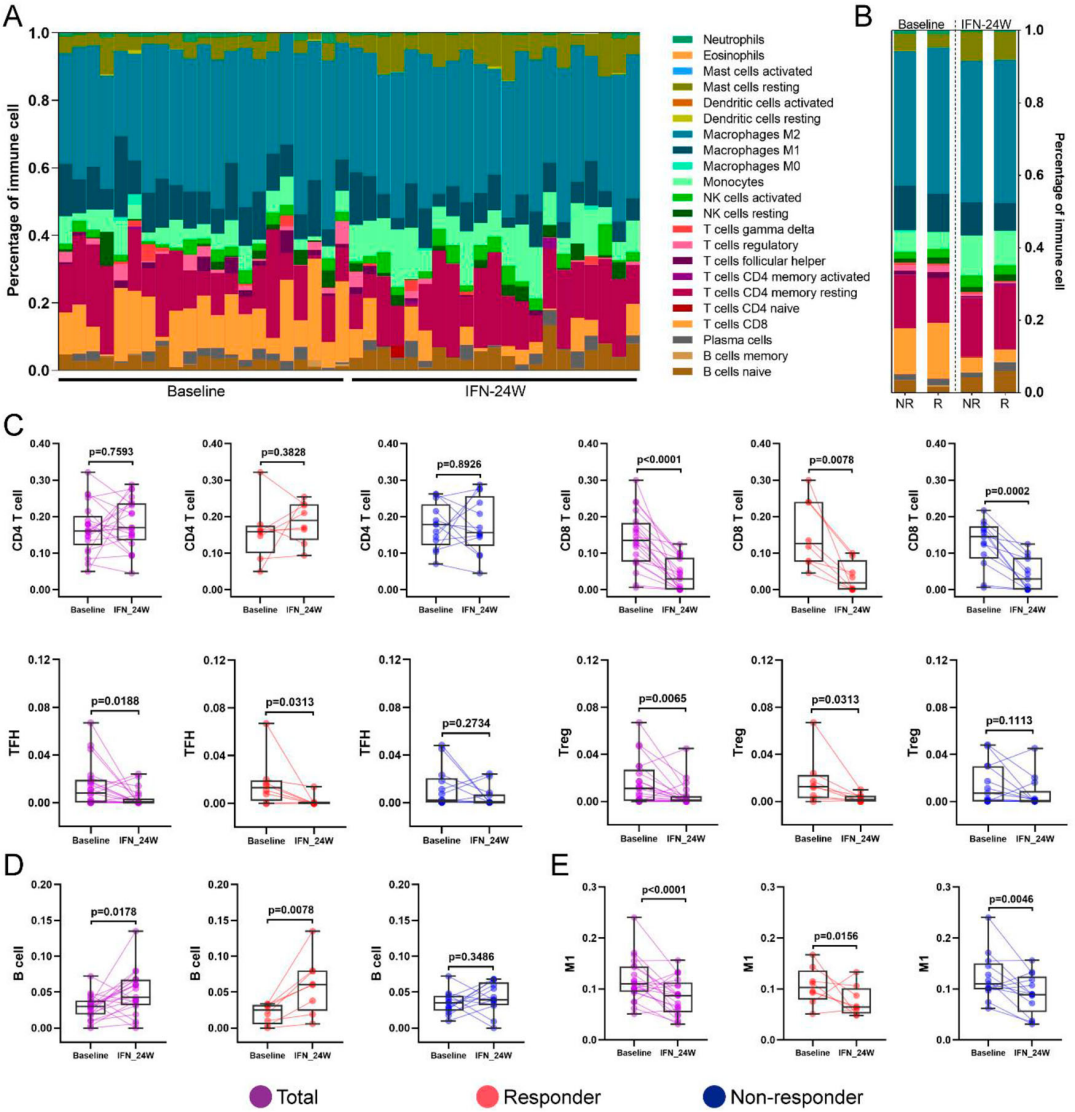
Immune cells infiltration analysis. (A) CIBERSORT was engaged to infer the proportion of infiltrated immune cells, the landscape of relative immune cells composition at baseline and IFN-24W across patients is shown. (B) Summary of relative immune cells composition at baseline and IFN-24W in responders and non-responders. (C) Alteration of total CD4 T cells was not significant in both responders and non-responders, a decrease of CD8 T cells was observed in both responders and non-responders, and a reduction of TFH and Treg cells was observed in responders rather than non-responders. (D) An increase of B cells was observed in responders. (E) A decrease of M1 macrophages was observed in both responders and non-responders. R, responder; NR, non-responder.

Immune cell infiltration in HBV-infected liver is an important part of antiviral immune response [19]. To comprehensively understand the compositional differences in infiltrated immune cells and their relationships to treatment response, we engaged a method known as CIBERSORT. Overview of immune cell fractions across samples is presented in Figure 6(A), which revealed that macrophage (particularly anti-inflammatory M2 population) was the predominant immune cell fraction, followed by T cell and monocyte, without any bias in responders or non-responders, at baseline or 24-week PegIFNα treatment (Figure 6(B)). No marked alteration of CD4 T cell was observed in responders or non-responders, while a sharp decrease of CD8 T cell was observed in both responders and non-responders (Figure 6(C)). In addition, a significant reduction of follicular helper T cell (TFH) and Treg was observed in responders compared to non-responders (Figure 6(C)). For B cells, a significant increase was observed in responders (Figure 6(D)). Although the proportion of total macrophage had no significant change in response to PegIFNα (Supporting Fig. S2A), M1 macrophage, rather than M2 macrophage, significantly decreased in responders and non-responders after 24-week PegIFNα treatment (Figure 6(E); Supporting Fig. S2B). In addition, an increase of monocyte and mast cell was observed in response to PegIFNα treatment (Supporting Fig. S2C, D), while there was no significant alteration of NK cells (Supporting Fig. S2E).

### Identification of meta-modules related to PegIFNα treatment

**Figure 7.**
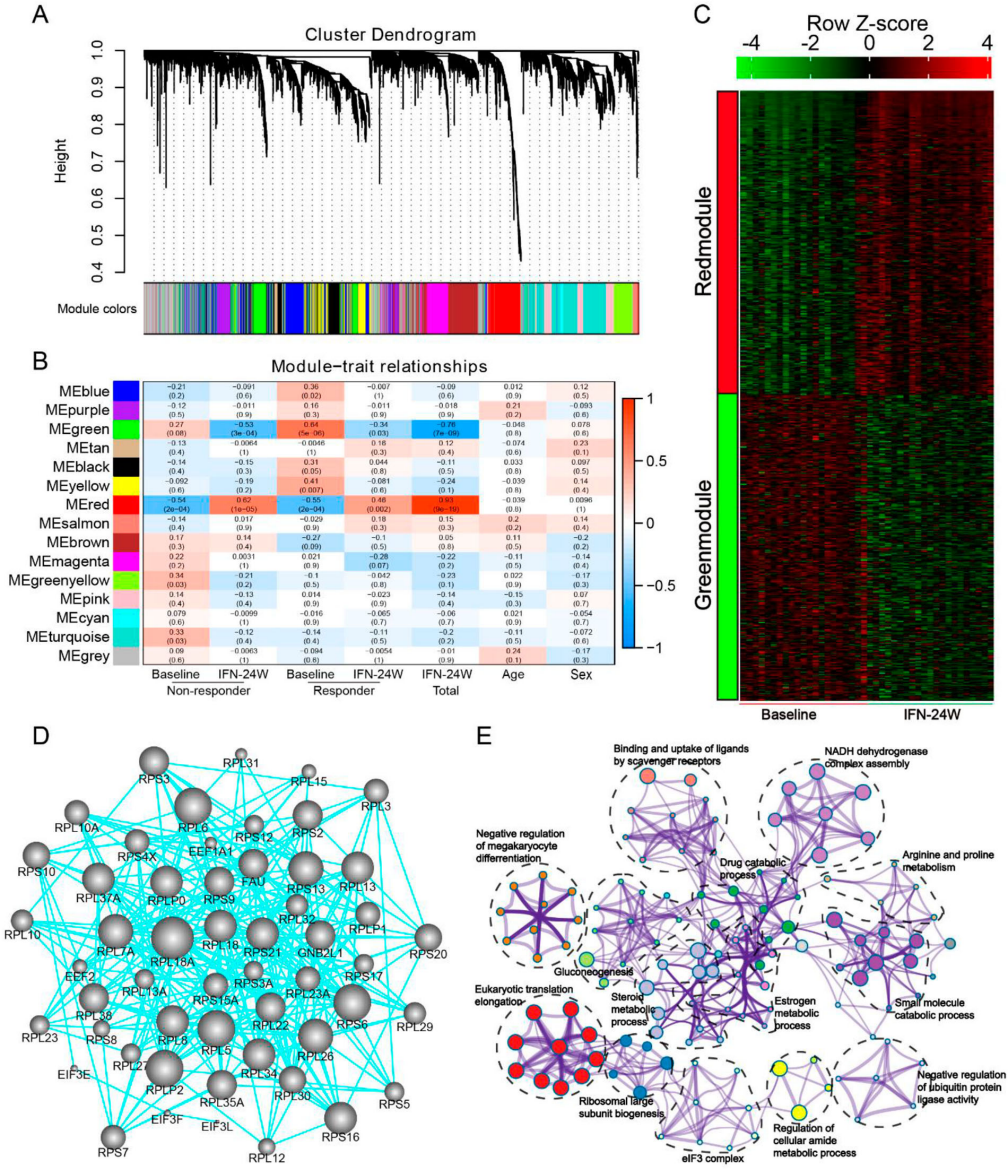
Identification of meta-modules related to PegIFNα treatment. (A) Cluster dendrogram of WGCNA analysis indicating expression of distinct gene modules. (B) Module-trait associations revealed that the green module was negatively correlated with treatment response, while the red module was positively correlated with PegIFNα treatment response. (C) Heatmap showed the expression of all genes in red and green modules at baseline and IFN-24W. (D) Genes in the green module were subjected to the STRING database to evaluate their hubness and the top 250 gene–gene interaction was displayed. Size of the dots represents hubness. (E) Functional enrichment analysis of genes in the green module demonstrated that these genes were enriched in pathway clusters including eukaryotic translation elongation, NADH dehydrogenase complex assembly, small molecule catabolic process, steroid metabolic process, etc. Different colours represent different clusters, and the size of dots represents enrichment significance.

In order to determine gene expression signatures correlated to PegIFNα treatment, weighted gene co-expression network analysis (WGCNA) was applied to the RNA-seq data. The results show that a total of 18 gene modules were identified (Figure 7(A)), and the Module Eigengene (ME), which served as a representative of a module, was calculated. Correlation between the ME values and several variables was evaluated, which shows that the red and green module are positively and negatively correlated with IFNα treatment, respectively (Figure 7(B)). Heatmap shows expression of all genes in each of the two modules across all samples at baseline and 24-week IFNα treatment (Figure 7(C)). Gene-gene network analysis of green module identified ribosomal proteins including RPL18A, RPL6, RPS6 and RPL5 as hub genes (Figure 7(D)). Functional enrichment analysis of genes in green module demonstrates that these genes are implicated in multiple biological processes including eukaryotic translation elongation, NADH dehydrogenase complex assembly and small molecule catabolic process (Figure 7(E)).

### Impact of HBeAg status on IFN treatment response

**Figure 8.**
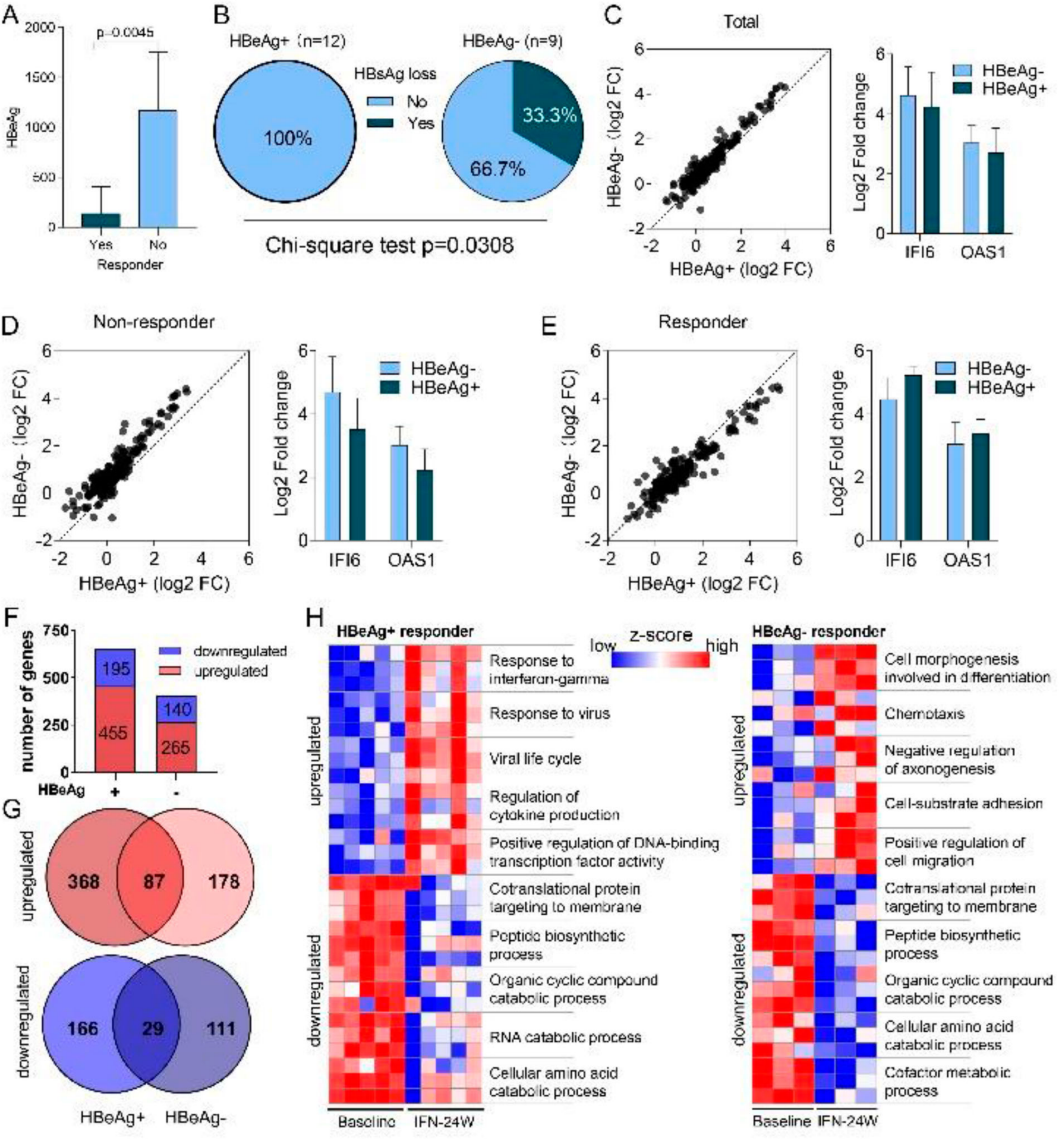
Impact of HBeAg status on PegIFNα treatment response. (A) Comparison between HBeAg+ responders and non-responders showed higher HBeAg levels in non-responders. (B) Higher HBsAg loss rate was achieved in HBeAg− patients. (C) Generally, fold change of ISGs was greater in HBeAg− patients than in HBeAg+ patients. (D) In non-responders, ISGs were upregulated more significantly in HBeAg− patients. (E) In responders, higher fold change of ISGs was observed in HBeAg+ patients. (F) Expression alteration of genes was analysed separately in HBeAg+ responders and HBeAg− responders, 455 genes were up-regulated and 195 genes were down-regulated in HBeAg+ responders, while 265 genes were up-regulated and 140 genes were down-regulated in HBeAg− responders. (G) Venn diagram shows that 368 and 178 genes were exclusively up-regulated in HBeAg+ and HBeAg− responders, respectively; while 166 and 111 genes were exclusively down-regulated in HBeAg+ and HBeAg− responders, respectively. (H) The exclusively dysregulated genes were subjected to GO term enrichment analysis, and heatmap shows *z* score transformed gene expression for top enriched biological processes. FC, fold change.

HBeAg is a secreted form of the viral precore protein and the exact function of HBeAg in HBV life cycle remains poorly understood [20]. Clinical studies have shown that CHB patients with positive HBeAg (HBeAg+) respond less effectively to type I IFN treatment than those with negative HBeAg (HBeAg−) [21,22]. Consistently, we here revealed that non-responders possessed higher baseline HBeAg levels than responders in HBeAg+ group (Figure 8(A)). More importantly, higher HBsAg loss rate was observed in the HBeAg− group compared to the HBeAg+ group (3/9 vs 0/12) (Figure 8(B)). We have recently reported that the cytoplasmic precursor of HBeAg, namely p22, blocks nuclear translocation of pSTAT1, thus impeding JAK-STAT signalling and reducing ISG expression to confer resistance to IFN therapy [23]. Therefore, we compared expression alterations of ISGs between HBeAg+ and HBeAg− patients, and found that most ISGs experienced greater fold change in HBeAg− patients in response to PegIFNα (Figure 8(C)). When treatment response was taken into consideration, a similar trend was observed in the non-responder group (Figure 8(D)), while in the responder group, most ISGs upregulated more significantly in HBeAg+ patients (Figure 8(E)). We then compared gene expression difference between HBeAg+ and HBeAg− responders. The results showed that according to the threshold mentioned above, 455 up-regulated and 195 down-regulated genes were identified in HBeAg+ responders, while 265 up-regulated and 140 down-regulated genes were identified in HBeAg− responders (Figure 8(F); Supporting Fig. S3A), and the top 15 up-regulated and down-regulated genes in each group are presented in Supporting Fig. S3B. Further analysis revealed that 368 and 178 genes were exclusively up-regulated in HBeAg+ and HBeAg− group, respectively (Figure 8(G)), while 166 genes were down-regulated uniquely in the HBeAg+ group and 111 genes were down-regulated uniquely in the HBeAg− group (Figure 8(G)). Functional enrichment analysis of these genes demonstrates that in the HBeAg+ group, up-regulated genes are mainly implicated in antiviral responses, while down-regulated genes are involved in viral gene expression and protein targeting to the ER membrane (Supporting Fig. S3C). In the HBeAg− group, up-regulated genes are significantly enriched in cell morphogenesis; while similar to the HBeAg+ group, down-regulated genes in the HBeAg− group are also enriched in viral gene expression and protein targeting to the ER membrane (Supporting Fig. S3C). Genes involved in top enriched GO biological processes are presented in Figure 8(H). We also analysed immune cell infiltration and found a decreased infiltration of CD8 T cell and T follicular helper cell and an increase of mast cells in both HBeAg+ and HBeAg− groups (Supporting Fig. S3D, E), however, the difference in HBeAg+ group was not statistically significant (Supporting Fig. S3F). In addition, although not statistically significant, an increase of naive B cell was observed in both HBeAg+ and HBeAg− group (Supporting Fig. S3F).

## Discussion

The PegIFNα is one of the approved therapies for chronic HBV infection, which possesses both antiviral and immunomodulatory effects, but the molecular mechanism of treatment response remains poorly understood [5]. To this aim, we herein analysed the intrahepatic gene expression profiles in paired liver biopsy samples obtained from 21 CHB patients before and at 24 weeks of PegIFNα treatment. For the first time, gene alteration and immune cell infiltration were evaluated and compared between responders and non-responders using baseline and on-treatment paired human liver biopsy samples (Figure 1). The current study thus provides novel insights into the antiviral mechanisms of PegIFNα in vivo.

The pleiotropic antiviral effects of IFNα on HBV replication have been demonstrated by numerous studies, which include but not limited to inhibiting HBV viral gene transcription or inducing cccDNA degradation [24–26], disrupting the assembly of pgRNA-containing capsids or promoting their degradation [27–29]. Our results revealed that PegIFNα administration down-regulated more genes than the number of up-regulated ones in CHB patient livers, but the up-regulated genes experienced more significant extent of alteration (Figure 2(A–C)). Functional enrichment analysis demonstrated that PegIFNα induced strong activation of antiviral signalling including responses to IFNα and defense responses to virus (Figure 2(D); Figure 3(A–D)). Generally, type I IFNs exert their antiviral effects by inducing expression of hundreds of ISGs [17]. In chronic hepatitis C (CHC) patients, it has been shown that PegIFNα treatment induced significant upregulation of ∼250 genes, of which most were ISGs [6]. We evaluated the expression of these genes in the current study and found 190 out of 250 genes were detectable. The results showed that 76 genes were up-regulated, 6 genes were down-regulated, while up to 108 genes showed no significant alteration (Figure 4(A)), which is quite different from findings in CHC. In addition, in CHC patients, investigators found that non-responders had higher expression levels of ISGs before treatment, and PegIFNα almost failed to further increase their expressions [6]. We here showed that in CHB patients, lower baseline levels of ISG expression were also observed in responders (Figure 4(D,E)), but PegIFNα promoted ISG expression in both responders and non-responders to a comparable level after 24-week IFNα treatment (Figure 4(E); Supporting Fig. S1A, B). Furthermore, when treatment response is taken into consideration, the dysregulation of the 190 genes was significantly different between responders and non-responders, and more genes were up-regulated in responders (Figure 4(C)). Such discrepancy between CHC and CHB is likely due to virus-specific mechanism(s), as HBV acts more like a stealth virus under the host innate surveillance [30]. A previous study using the woodchuck hepatitis virus (WHV) model revealed that the antiviral response to woodchuck IFN-alpha did not correlate with the intrahepatic induction of ISGs [9]. However, our results obtained from clinical samples indicate that a favourable PegIFNα treatment response indeed depends on the significant upregulation of a large number of ISGs, rather than a few specific ISGs (Figure 4(B)). Hence, it is worth noting that, for the above studies, both the host-and virus-specific regulations of IFN treatment response should be taken into consideration in terms of data analyses and interpretations.

Immunomodulatory effect is another mechanism by which IFNα suppresses HBV infection. We engaged CIBERSORT to assess the impact of PegIFNα treatment on intrahepatic immune cell infiltration (Figure 6(A,B)). Our data showed that the proportion of B cells was enlarged only in responders (Figure 6(D)), while infiltration of monocyte and mast cell was increased in both responders and non-responders after PegIFNα treatment (Supporting Fig. S2C, D). In addition, T cell infiltration and M1 macrophage were decreased in both responders and non-responders (Figure 6(C,E)). Consistent with our CIBERSORT results, a recent study demonstrated a similar alteration of T cell phenotypic profiles in peripheral blood during HBsAg loss [31]. The infiltrated immune cells in livers of responders and non-responders can be more quantitatively analysed by flow cytometry and/or single cell RNA sequencing in future study.

An effective T cell immune response contributes to HBV clearance, but was usually impaired during chronic HBV infection [32]. Type I IFN has been reported to suppress T cell activation by promoting PD1 expression and blocking PD1 pathway can restore the antiviral T-cell response in CHB [33–35]. Besides, Kupffer cells in liver can support HBV-induced CD8 T cell exhaustion [36]. In agreement with previous studies [10], here we also demonstrated that CD8 T cell infiltration was decreased in both responders and non-responders under PegIFNα administration (Figure 6(C)). This is consistent with functional enrichment analysis of down-regulated genes showing that T cell activation and adaptive immune response are inhibited by PegIFNα (Figure 2(D)), which may ameliorate liver inflammation and tissue damage.

The B cell-mediated humoral immune response plays an important role in limiting HBV infection [37], which may in turn affect the function of specific subset of B cell. It has been reported that HBsAg-specific B cell in CHB patients showed atypical memory phenotype and damaged classical memory B cell [38]. This kind of HBsAg-specific B cell showed impaired antibody production and functional exhaustion, which could be partially rescued by PD-1 blockade [39,40]. In addition, a recent study revealed that HBcAg-specific B cell presented classical memory phenotype and their response was associated with natural history of HBV infection [41]. In line with this, type I IFN treatment has been reported to increase B cell activity by directing B cells into transitional, regulatory population in multiple sclerosis patients [42]. However, a latest study reported that treatment with tenofovir disoproxil fumarate plus PegIFNα reduced B cell infiltration in CHB patients [43]. Thus, whether PegIFNα treatment can improve B cell function and increase antibody production in CHB deserves further investigation.

Macrophage can be polarized to a pro-inflammatory M1 type which involved in pathogen clearance or an anti-inflammatory M2 type implicating in efferocytosis [44]. Bility et al. demonstrated that HBV promoted M2-like activation in macrophage and thus led to HBV-induced immune impairment and liver disease in a humanized mouse model [45]. We here found that PegIFNα treatment decreased infiltration of M1 macrophages without affecting M2 macrophage counts but increased monocytes in both responders and non-responders (Figure 6(E); Supporting Fig. S2B, C). The results indicate that PegIFNα repressed intrahepatic monocyte differentiation into M1 macrophage, in CHB patients, hence, M1 macrophage is not involved in IFN-mediated antiviral response.

As for monocytic population, previous studies have demonstrated that HBV infection resulted in immunosuppressive monocytes expansion and led to T cell dysfunction [46–48]. In addition, it has also been reported that IFNα could enhance the cytotoxicity of monocytes [49]. Our data showed that PegIFNα treatment increased monocytes infiltration (Supporting Fig. S2C). We are yet to decipher if these monocytes are immunosuppressive or cytotoxic to HBV-infected hepatocytes.

Type I IFN is known to suppress mast cell function [50]. However, it has been reported that in CHC, type I IFN could enhance the secretion of mast cell-derived HLA-G, which was associated with liver fibrosis [51,52], indicating that type I IFN may promote HCV-related liver fibrosis, which seems contrary to the current evidence that IFN acts against liver fibrosis [53]. Here we found that PegIFNα treatment was associated with increased mast cell infiltration (Supporting Fig. S2D), the findings need further confirmation and the potential function of these mast cells should be assessed in future study.

It has been reported that IFNα can expand and activate functional NK cell population, resulting in an enhanced antiviral response [54,55]. A previous study with the woodchuck model showed that intrahepatic NK cells were increased by IFNα treatment and inhibited WHV via both cytolytic and non-cytolytic mechanisms [9]. However, no significant alteration of NK cell infiltration was observed in our study (Supporting Fig. S2E). In this regard, it is also worth noting that the regulation of intrahepatic NK cell by IFNα can be species-specific [11].

We employed WGCNA to identify gene signatures highly correlated with PegIFNα treatment. Fifteen modules were determined and the red module was significantly positively associated with PegIFNα treatment, while the green module showed significant negative correlation with PegIFNα treatment (Figure 7(A–C)). Gene–gene network analysis identified RPL18A, RPL6 and RPS6 as hub genes of the green module (Figure 7(D)). Functional enrichment analysis revealed that genes in the green module were mainly implicated in eukaryotic translation elongation, NADH dehydrogenase complex assembly and small molecule catabolic process (Figure 7(E)). RPL18A has been implicated in viral gene transcription. It has been previously observed that during dengue virus infection, RPL18 is redistributed to the perinuclear region and RPL18 inactivation results in reduced viral translation and replication [56]. RPL6 is involved in MHC class I antigen presentation and enables T cell immune surveillance of viruses, and the depletion of RPL6 has been shown to decrease the generation of influenza A virus-encoded peptide and impair ubiquitin-dependent peptide presentation [57]. In HCV-infected cells, RPS6 played a critical role in facilitating HCV translation over host translation [58]. The roles of these ribosomal proteins in regulating HBV replication deserve further exploration.

We have recently revealed that the intracellular HBeAg cripples IFNα signalling to desensitize IFN therapy [23], and thus we also investigated the differences between HBeAg+ and HBeAg− responders in the current study. We observed lower HBeAg levels in responders than non-responders in HBeAg+ patients and higher HBsAg loss rate in HBeAg− patients than HBeAg+ patients (Figure 8(A,B)). In addition, in all patients and non-responders, greater fold change of ISGs was observed in HBeAg− patients (Figure 8(C–D)). These observations are in accordance with previous studies showing a negative impact of HBeAg on patient’s response to IFN therapy [21–23]. Interestingly, in responders, ISGs were upregulated more significantly in a limited number of HBeAg+ patients than HBeAg− patients (Figure 8(E)), indicating that a higher level of ISG induction is required for HBeAg+ patients to become responders (HBeAg seroconversion), although the underlying mechanism is unclear. We further revealed that more genes dysregulated in HBeAg+ patients than HBeAg− patients, and most of the dysregulated genes were unique between the two groups (Figure 8(A,B)). Functional enrichment analyses indicated that genes exclusively up-regulated in HBeAg+ patients were mainly implicated in antiviral responses, while genes exclusively up-regulated in the HBeAg− group were enriched in cell morphogenesis (Supporting Fig. S3C). Interestingly, although genes exclusively down-regulated in HBeAg+ and HBeAg− groups were different, but both were enriched in biological processes including viral gene expression and protein targeting to the ER membrane (Supporting Fig. S3C), indicating that IFNα may specifically influence the production of HBeAg and/or HBsAg, as both viral antigens require ER for expression and secretion [59]. Interestingly, a group of down-regulated genes are related to the signal recognition particle (SRP)-dependent cotranslational protein targeting to membrane (Supporting Fig. S3C), inferring that the biosynthesis of the precore protein and subsequent HBeAg may be inhibited by IFNα treatment. Immune cell infiltration analysis indicated that although the differences in HBeAg+ group were not statistically significant, naive B cell and mast were increased, while CD8 T cell and T follicular helper cell were decreased in HBeAg+ and HBeAg− group as above described (Supporting Fig. S3F; Figure 6(C)). PegIFNα treatment up-regulated cell morphogenesis-related genes in HBeAg− CHB patients, but whether and how cell morphogenesis impact IFN-mediated anti-HBV response awaits further investigation.

In summary, by exploring the alteration of intrahepatic gene expression profile in CHB patients who received PegIFNα treatment, we demonstrated that responders had a higher upregulation magnitude of certain antiviral ISGs. In addition, we found various pathways and immune cells were associated with IFNα treatment response. Furthermore, ribosomal proteins and ER-associated proteins were identified as critical players in antiviral response. These findings enriched our understanding of IFN-mediated anti-HBV effects and provided novel insights into the development of potential strategies to improve IFNα therapy.

## Supplementary Material

Supplemental Material
