# Supplementary material for: Intrahepatic transcriptomics reveals gene signatures in chronic hepatitis B patients responded to interferon therapy
Source: Emerg Microbes Infect. 2022 Jul 27;11(1):1876–89. doi: 10.1080/22221751.2022.2100831 (PMC9336496; doi:10.1080/22221751.2022.2100831)
Supplement: Supplemental Material [file TEMI_A_2100831_SM2106.zip › Supplementary_Materials_and_Figures.docx]

**Supplemental Materials & Methods**

***Histological analysis***

Fibrosis stage and necroinflammatory activity were scored using Scheuer classification system by Department of Pathology, Huashan Hospital.

***RNA sequencing and data analyses***

RNA was extracted from frozen liver biopsy tissues using the TRIzol reagent as per a standard protocol [[1](#_ENREF_1)]. The RNA samples were sequenced using Illumina Hiseq3000. The raw reads were firstly trimmed using the program trim_galore (v0.6.4) with parameters: “–paired –illumine”, to remove low quality reads and adapter reads. We then mapped the trimmed reads to the human reference genome (UCSC hg19) using the software TopHat (v2.1.1) with default parameters. The program Cufflinks (v2.2.1) with default parameters was used to assign the mapped reads to human transcript annotation (UCSC hg19) to identify the gene expression abundance, which were represented by FPKM (Fragments Per Kilobase of transcript per Million mapped reads). The FPKM values were normalized to TPM (Transcripts Per Kilobase Million) using the script, FPKM2TPM.R in website (<https://github.com/FeizhenWu/Nono>). The normalization allows the comparison of gene expression between samples. The program cuffdiff with default parameters in the Cufflinks suite was used to calculate the fold-change and p-value of genes for comparison between groups. The clusterProfiler (v3.14.0) was used to perform GO term and KEGG pathway enrichment analysis for differential expression genes [[2](#_ENREF_2)]. We performed the gene set enrichment analysis (GSEA) for gene list with fold-change.

***Immune cell estimation***

To identify the abundance of immune cells in these biopsies, we used the TPM from the RNA-seq analysis. According to TPM values, we calculated the abundance of immune cells by using the CIBERSORT method [[3](#_ENREF_3)].

***WGCNA analysis***

Co-expression network was built using the WGCNA package in R. Genes were selected with TPM>=1 in each sample, and then decreasingly sorted according to median absolute deviation. The top 5000 genes of robust expression in the sorted list were included in the network. We firstly chosen 18 as a soft-thresholding power using the pickSoftThreshold function. Network construction was performed using the blockwiseModules function with parameters: power=18, networkType = "signed", deepSplit = 2, maxBlockSize = 6000, TOMType = "signed", corType = "bicor", minModuleSize = 30, reassignThreshold = 0, and mergeCutHeight = 0.25, which allows the network construction and module detection for the entire data set. Genes were assigned into their corresponding modules. Each module was summarized by the first principal component of the TPMs of genes in the module. Thus, the module eigengene represents the maximum amount of variation of the module expression levels. Eigengene of each module was calculated with the moduleEigengenes function. The Pearson correlation between eigengenes and classifications (trait) of samples were calculated using correlation function. The corPvalueStudent function were used to calculate student asymptotic p-value for the correlations. Thus, genes were assigned into a corresponding module, and the correlation between modules and traits were calculated. The correlation heatmap was plotted with the labeled Heatmap function.

**References**

[1] Rio DC, Ares M, Jr., Hannon GJ, et al. Purification of RNA using TRIzol (TRI reagent). Cold Spring Harbor protocols. 2010;2010:pdb prot5439.

[2] Yu G, Wang LG, Han Y, et al. clusterProfiler: an R package for comparing biological themes among gene clusters. Omics : a journal of integrative biology. 2012;16:284-7.

[3] Newman AM, Liu CL, Green MR, et al. Robust enumeration of cell subsets from tissue expression profiles. Nat Methods. 2015;12:453-7.

**Supplementary Table 1.** Demographic and clinical characteristics of the included patients.

| **ID** | **Sex** | **Age** | **ALT (IU/mL)**  BL/W24 | **HBeAg**  BL/W24 | **HBsAg (log10)**  BL/W24 | **HBV DNA (log10)**  BL/W24 | **Grade**  BL/W24 | **Stage**  BL/W24 | **HBV DNA**  U.D. | **Sero-conversion** | **Response** |
| --- | --- | --- | --- | --- | --- | --- | --- | --- | --- | --- | --- |
| 1 | M | 33 | 290/106 | +/+ | 4.48/4.33 | 7.48/7.43 | 3/4 | 2/4 | W48 | No | NR |
| 2 | M | 27 | 41/31 | +/- | 3.29/3.41 | 6.24/4.74 | 2/1 | 2/3 | W48 | W24 | R |
| 3 | M | 30 | 118/78 | -/- | 3.51/3.46 | 5.27/Neg. | 3/2 | 3/2 | W24 | No | NR |
| 4 | F | 25 | 82/42 | +/+ | 4.72/4.66 | 7.70/7.32 | 1/2 | 1/1 | W72 | No | NR |
| 5 | M | 43 | 159/75 | +/+ | 4.72/4.41 | 7.48/7.21 | 2/2 | 0/2 | W48 | No | NR |
| 6 | F | 36 | 98/20 | +/- | 3.48/0.91 | 6.68/Neg. | 2/1 | 2/1 | W24 | W24 | R |
| 7 | M | 31 | 64/154 | +/+ | 4.56/3.37 | 7.30/5.10 | 3/2 | 3/3 | W48 | W48 | R |
| 8 | M | 23 | 178/46 | +/+ | 4.02/4.07 | 6.78/6.20 | 2/2 | 0/1 | W48 | No | NR |
| 9 | M | 28 | 132/31 | +/+ | 3.96/4.22 | 7.16/3.42 | 2/1 | 2/2 | W48 | W48 | R |
| 10 | M | 27 | 156/29 | -/- | 3.22/1.49 | 6.07/Neg. | 2/2 | 2/1 | W24 | W48 | R |
| 11 | F | 56 | 110/51 | -/- | 2.91/2.84 | 4.59/Neg. | 2/2 | 2/2 | W24 | No | NR |
| 12 | M | 57 | 474/24 | -/- | 3.24/2.78 | Neg./Neg. | 3/2 | 2/2 | W0 | No | NR |
| 13 | M | 30 | 31/90 | +/+ | 3.33/3.33 | 4.44/Neg. | 3/2 | 3/2 | W24 | W72 | R |
| 14 | F | 45 | 98/46 | -/- | 3.13/3.26 | 5.21/4.55 | 3/1 | 2/1 | W0 | No | NR |
| 15 | M | 25 | 107/69 | -/- | 3.08/2.61 | 7.05/Neg. | 2/2 | 2/2 | W24 | W48 | R |
| 16 | M | 25 | 79/105 | -/- | 1.75/Neg. | Neg./Neg. | 1/1 | 1/0 | W0 | W24 | R |
| 17 | M | 31 | 60/84 | -/- | 4.26/4.39 | 6.62/5.85 | 2/1 | 1/2 | W48 | No | NR |
| 18 | M | 27 | 214/205 | +/+ | 4.25/4.08 | 2.80/Neg. | 2/2 | 3/4 | W24 | No | NR |
| 19 | M | 26 | 678/49 | +/+ | 4.66/2.68 | 7.60/Neg. | 3/2 | 2/1 | W24 | No | NR |
| 20 | F | 26 | 268/87 | +/+ | 4.18/3.41 | 7.60/4.07 | 2/2 | 2/2 | W48 | No | NR |
| 21 | M | 50 | 18/54 | -/- | 2.75/1.92 | Neg./Neg. | 1/0 | 1/0 | W0 | No | NR |

M, male; F, female; BL, baseline; W, week; U.D., undetectable (<50IU/mL); Neg., negative; R, responder; NR, non-responder

**Supplementary Table 2.** Summary of anti-HBV ISGs

| **Gene Symbol** | **Reported Mechanism** | **Reference (PMID)** |
| --- | --- | --- |
| MX1 | Interacts with hepatitis B core antigen and inhibits nucleocytoplasmic export of viral mRNA | 11222692  22271421 |
| MYD88 | Activates NF-kappa B signaling pathway and downregulates viral transcription | 14980490 |
| A3G/A3F | Induces extensive G-to-A hypermutations in a fraction of the replicated HBV genomes; A3G also inhibits HBV replication in a deaminase-independent manner | 16729314  17314171 |
| ZAP | Inhibits HBV replication through posttranscriptional down-regulation of viral pgRNA | 23853601 |
| IFIT1/2 | Inhibits HBV replication at both transcriptional and posttranscriptional steps | 23867918 |
| TRIM5/6/11/14/  25/26/31/41 | Reduces HBV mRNA expression and inhibits HBV enhancer I / II activities | 23936368 |
| A3A | Induces extensive G-to-A hypermutations of HBV cccDNA for degradation | 24557838 |
| BST2 | Inhibits HBV virion secretion | 26109732 |
| TRIM25 | Promotes IFN production and reduces HBV replication | 28194021 |
| ISG20 | Degrades HBV RNA | 28399146 |
| IFI16 | May epigenetically inhibit cccDNA transcription | 31402464 |
| MX2 | Reduces HBV RNA levels and may also indirectly inhibit cccDNA formation | 31863794 |
| SAMD4A | Binds to the SRE site in viral RNA to trigger its degradation | 32341522 |

**
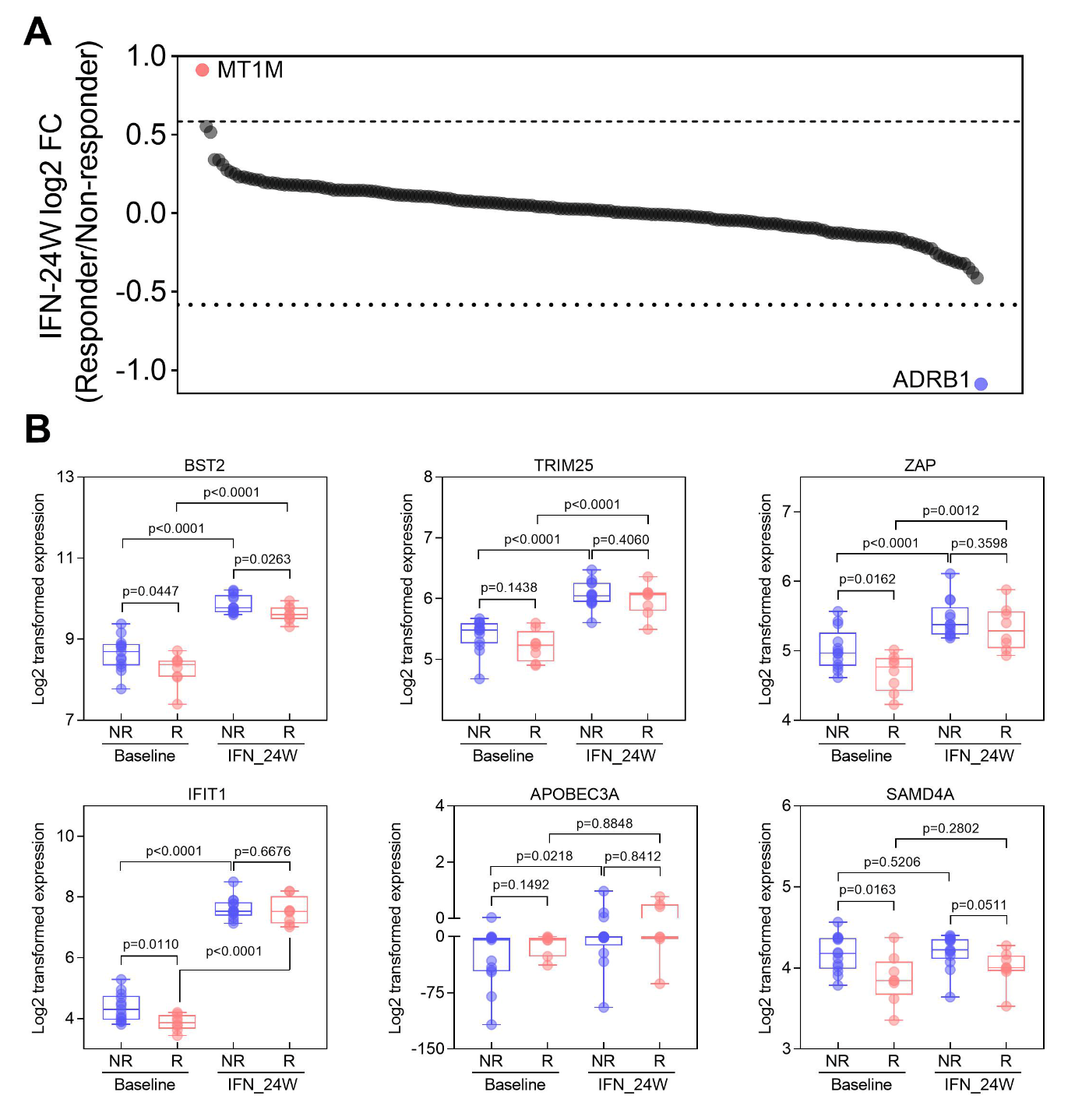
Supplementary Figures**

**Supplementary Figure 1. Antiviral ISGs had lower baseline levels and experienced higher fold change in responders.** (A) Analysis revealed comparable expression level of ISGs between responders and non-responders at IFN-24W on-treatment. (B) Expressions of selected anti-HBV ISGs at baseline and IFN-24W.


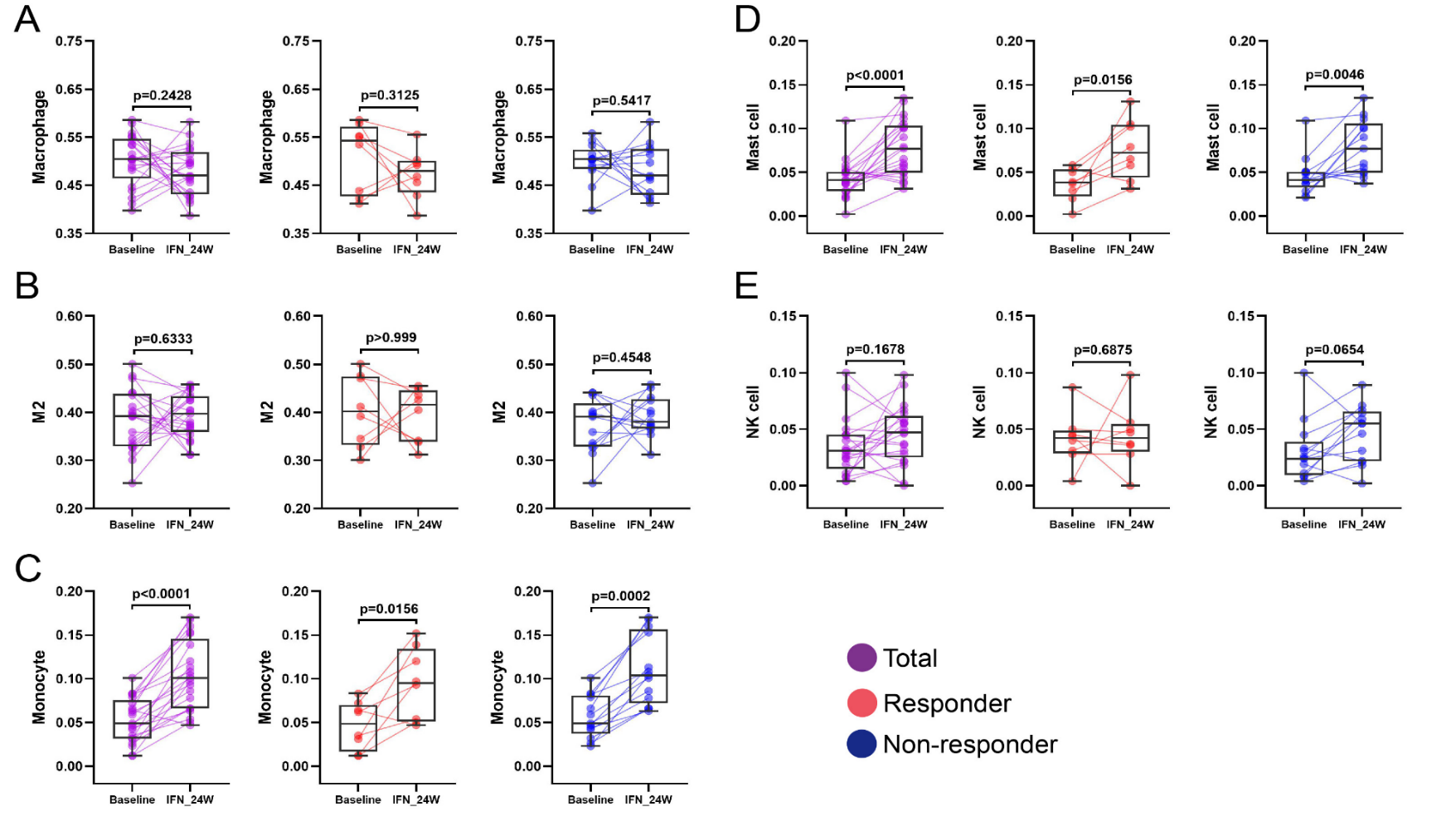

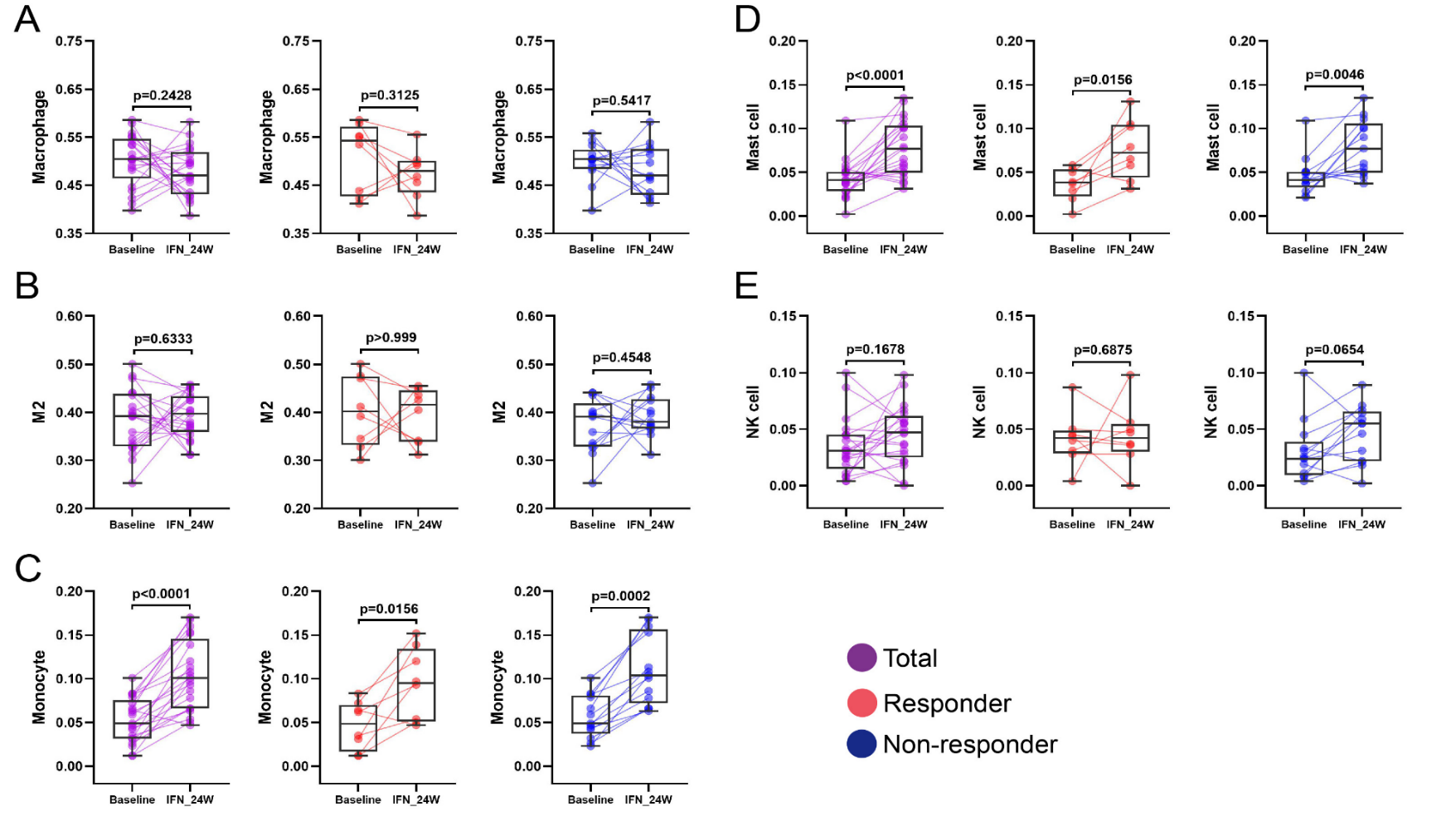

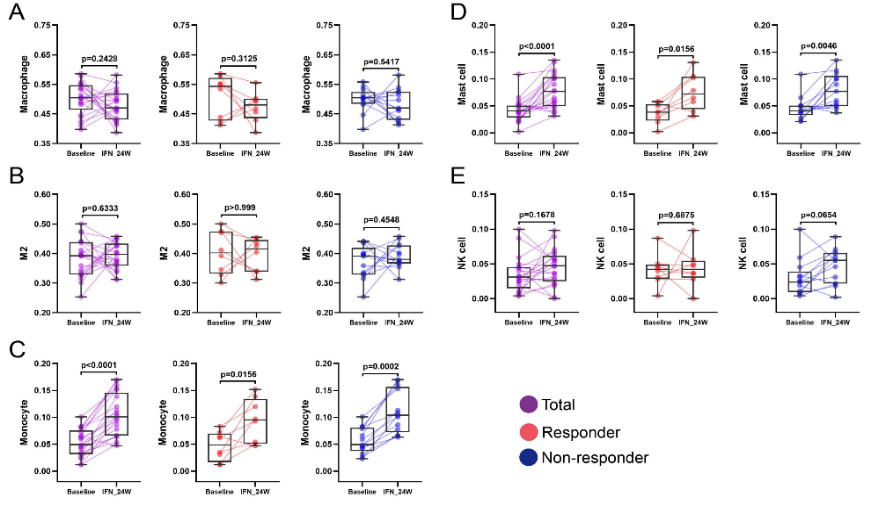


**Supplementary Figure 2. Immune cell infiltration analysis.** (A-B) The proportion of total macrophage and M2 macrophage showed no significant alteration. (C-D) PegIFNα treatment increased monocyte and mast cell infiltration. (E) Alteration of NK cell was not significant.

**
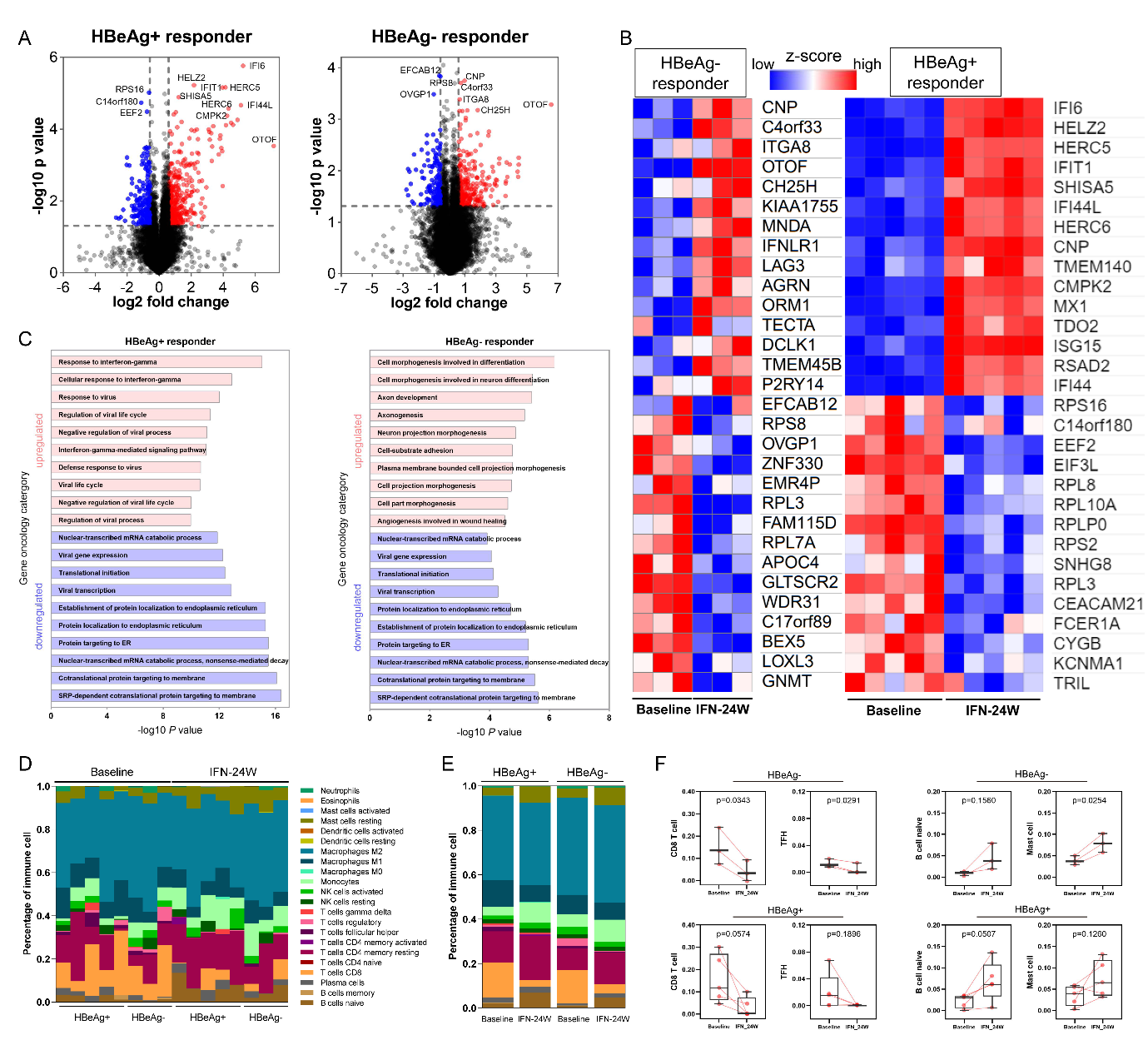
Supplementary Figure 3. Impact of HBeAg status on PegIFNα treatment response.** (A) Volcano plots showed genes differentially expressed in HBeAg+ and HBeAg- responders and name of selected top differentially expressed genes (based on significance) was labeled. (B) Heatmaps showed the top 15 up-regulated and down-regulated genes in each group. (C) GO enrichment analysis of genes exclusively up- or down-regulated in each group, top 10 enriched GO terms were presented. (D-E) Overview and summary of immune cell composition at baseline and IFN-24W. (F) Immune cell infiltration analysis showed that alteration of immune cell proportion in HBeAg- responder was in accordance with that in HBeAg+ responder. Decreased infiltration of CD8 T cell and TFH cell was observed at IFN-24W, while infiltration of naive B cell and mast cell were increased, although some alterations were not statistically significant.
