# Supplementary figures and images for: Intrahepatic transcriptomics reveals gene signatures in chronic hepatitis B patients responded to interferon therapy
Source: Emerg Microbes Infect. 2022 Jul 27;11(1):1876–89. doi: 10.1080/22221751.2022.2100831 (PMC9336496; doi:10.1080/22221751.2022.2100831)

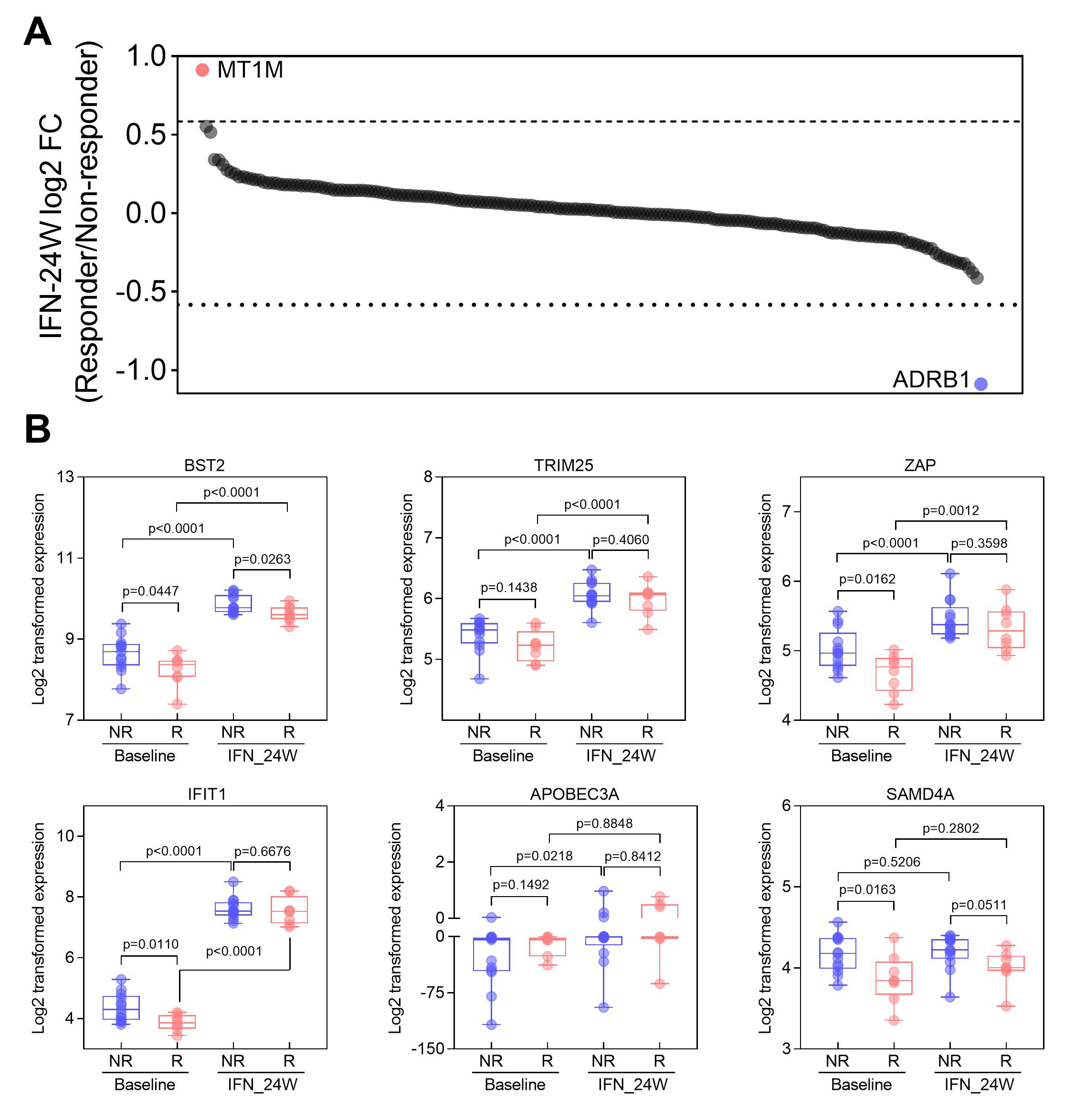

Supplement: Supplemental Material [file TEMI_A_2100831_SM2106.zip › Figure S1.tif]

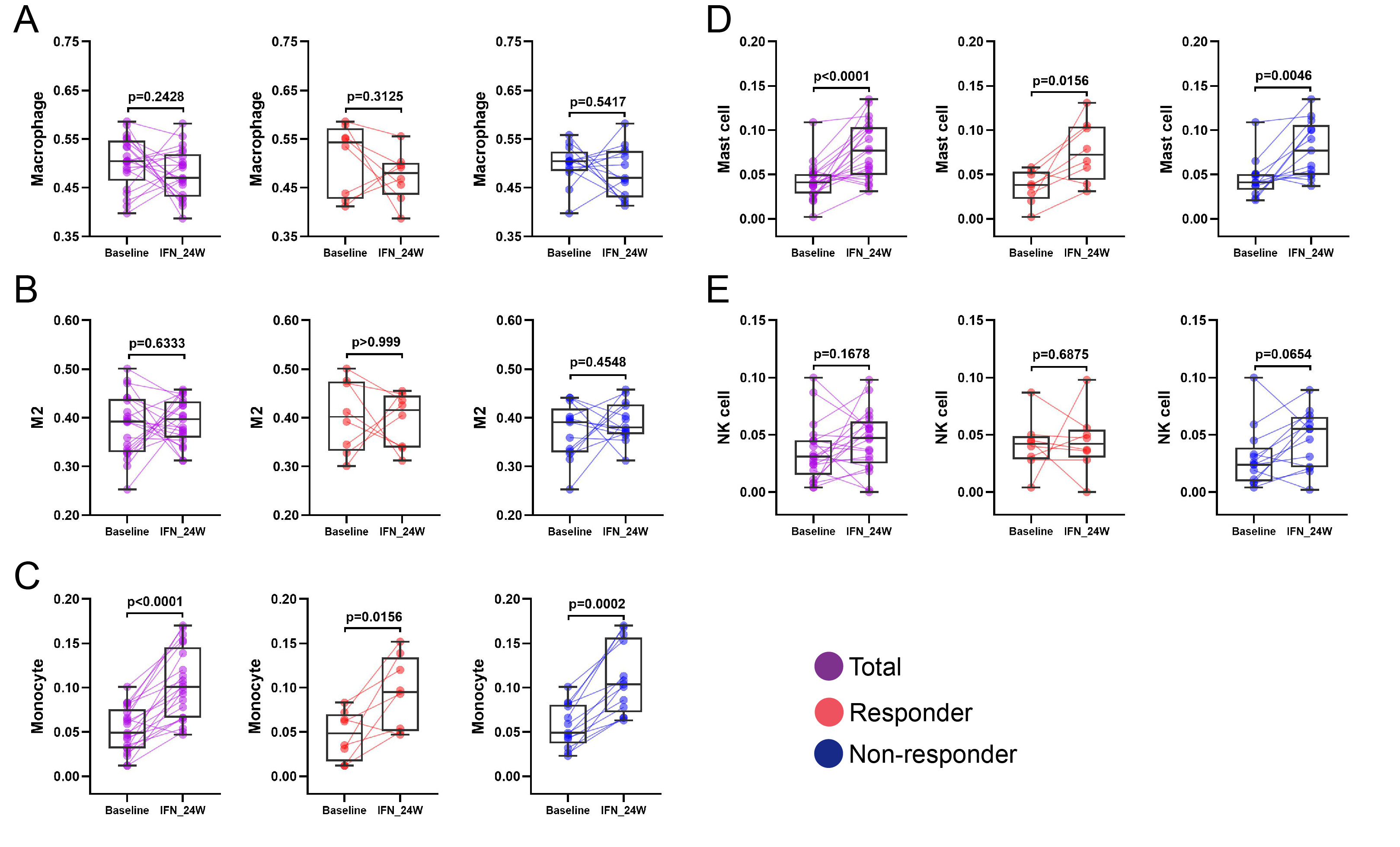

Supplement: Supplemental Material [file TEMI_A_2100831_SM2106.zip › Figure S2.tif]

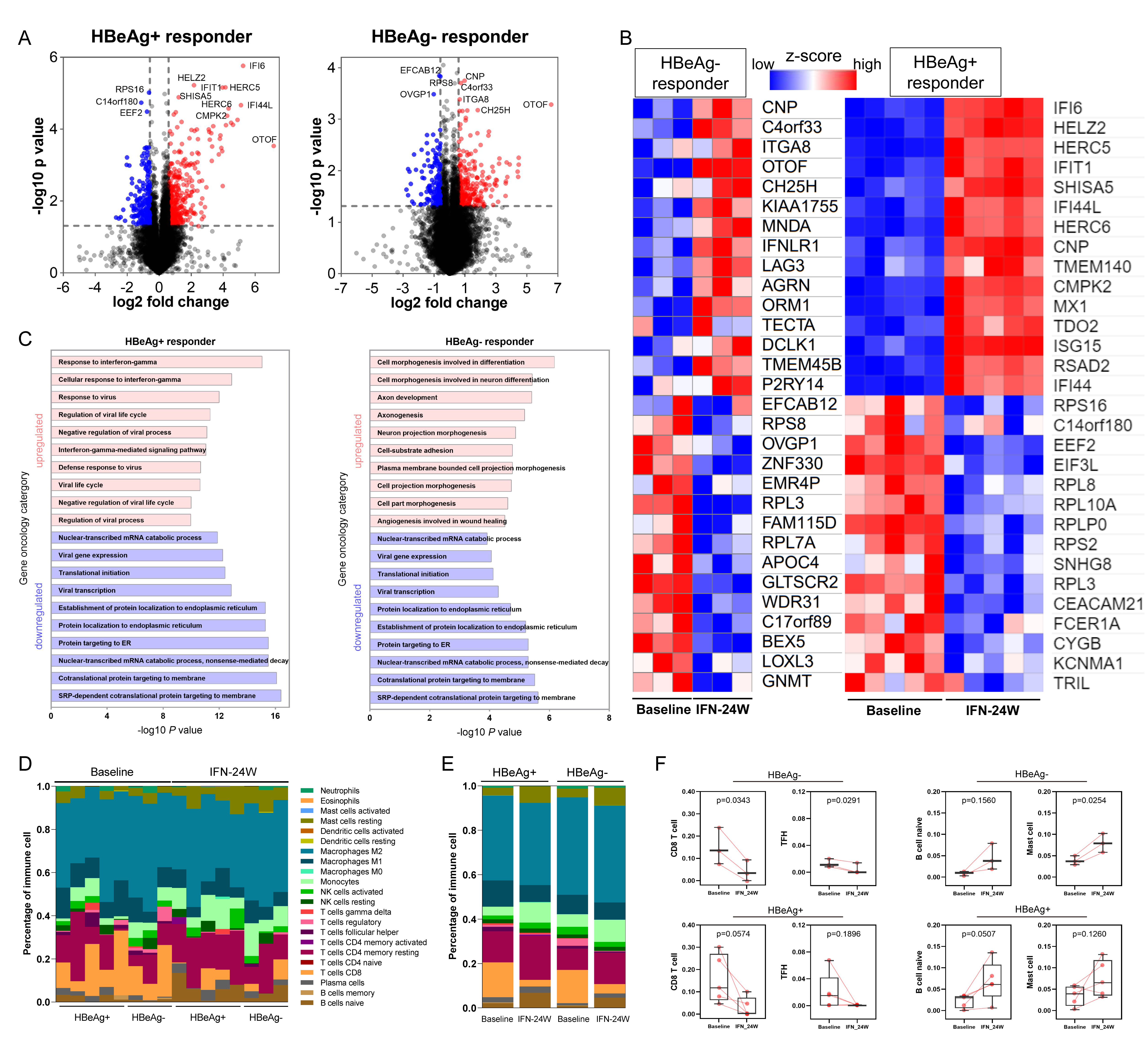

Supplement: Supplemental Material [file TEMI_A_2100831_SM2106.zip › Figure S3.tif]
